# Perilipin 1 Mediates Lipid Metabolism Homeostasis and Inhibits Inflammatory Cytokine Synthesis in Bovine Adipocytes

**DOI:** 10.3389/fimmu.2018.00467

**Published:** 2018-03-09

**Authors:** Shiqi Zhang, Guowen Liu, Chuang Xu, Lei Liu, Qiang Zhang, Qiushi Xu, Hongdou Jia, Xiaobing Li, Xinwei Li

**Affiliations:** ^1^Key Laboratory of Zoonosis, Ministry of Education, College of Veterinary Medicine, Jilin University, Changchun, Jilin, China; ^2^College of Animal Science and Veterinary Medicine, Heilongjiang Bayi Agricultural University, Daqing, China; ^3^College of Veterinary Medicine, Hunan Collaborative Innovation Center of Safety Production of Livestock and Poultry, Hunan Agricultural University, Changsha, China

**Keywords:** PLIN1, lipid synthesis, lipolysis, NF-κB inflammatory pathway, inflammation

## Abstract

Dairy cows with ketosis displayed lipid metabolic disorder and high inflammatory levels. Adipose tissue is an active lipid metabolism and endocrine tissue and is closely related to lipid metabolism homeostasis and inflammation. Perilipin 1 (PLIN1), an adipocyte-specific lipid-coated protein, may be involved in the above physiological function. The aim of this study is to investigate the role of PLIN1 in lipid metabolism regulation and inflammatory factor synthesis in cow adipocytes. The results showed that PLIN1 overexpression upregulated the expression of fatty acid and triglyceride (TAG) synthesis molecule sterol regulator element-binding protein-1c (SREBP-1c) and its target genes, diacylglycerol acyltransferase (DGAT) 1, and DGAT2, but inhibited the expression of lipolysis enzymes hormone-sensitive lipase (HSL) and CGI-58 for adipose triglyceride lipase (ATGL), thus augmenting the fatty acids and TAG synthesis and inhibiting lipolysis. Importantly, PLIN1 overexpression inhibited the activation of the NF-κB inflammatory pathway and decreased the expression and content of tumor necrosis factor alpha (TNF-α), interleukin 1 beta (IL-1β), and interleukin 6 (IL-6) induced by lipopolysaccharide. Conversely, PLIN1 silencing inhibited TAG synthesis, promoted lipolysis, and overinduced the activation of the NF-κB inflammatory pathway in cow adipocytes. In ketotic cows, the expression of PLIN1 was markedly decreased, whereas lipid mobilization, NF-κB pathway, and downstream inflammatory cytokines were overinduced in adipose tissue. Taken together, these results indicate that PLIN1 can maintain lipid metabolism homeostasis and inhibit the NF-κB inflammatory pathway in adipocytes. However, low levels of PLIN1 reduced the inhibitory effect on fat mobilization, NF-κB pathway, and inflammatory cytokine synthesis in ketotic cows.

## Introduction

The transition period is the most metabolically challenging period for dairy cattle and is characterized by homeostatic changes, negative energy balance (NEB), and increased risk for metabolic disorders (1, 2). Over 60% of all dairy cows develop ketosis or fatty liver during the transition period, which is a direct response to an energy imbalance and may decrease hepatic efficiency, animal health, and milk production (3, 4). Excessive NEB initiated fat mobilization of adipose tissues and a subsequent increase in blood concentrations of non-esterified fatty acid (NEFA) and ketone bodies (5). Therefore, the lipid metabolism of adipose tissue in cows with ketosis or fatty liver exhibited a disordered state. Adipose tissue, especially white adipose tissue, is the most important lipid storage site for animals (6). Lipid droplets (LDs) are monolayer organelles in the cytoplasm of adipocytes and are shown to be highly dynamic, playing a key role in maintaining energy homeostasis (7). There are many proteins that are coated on the surface of LDs. These proteins act as goalkeepers of LDs and messengers of cytoplasmic proteins interacting with other organelles in cells that control lipid homeostasis (8, 9). Perilipin 1 (PLIN1) is highly expressed in white adipocytes and is actively involved in the regulation of lipolysis through interaction with lipases [hormone-sensitive lipase (HSL)] and lipase activators [CGI-58 for adipose triglyceride lipase (ATGL)] (10, 11). A recent study has shown that PLIN1 also augments triglyceride (TAG) synthesis and promotes the enlargement of LDs, leading to the formation of large LDs (12, 13). However, the relevant mechanism of PLIN1 on the lipid metabolism of adipocytes is still not completely characterized.

Dairy cows with ketosis or fatty liver exhibit high levels of systemic inflammation (3, 14). Laboratory detection has demonstrated that the blood concentration of cytokines, including interleukin 1 beta (IL-1β), interleukin 6 (IL-6), and tumor necrosis factor alpha (TNF-α), was significantly increased in cows with ketosis or fatty liver compared with healthy cows (3, 14). White adipose tissue is no longer considered to be an inert tissue mainly devoted to energy storage, but is emerging as an active participant in regulating physiologic and pathologic processes, including immunity and inflammation (15). Adipose tissue produces and releases a variety of proinflammatory cytokines, such as TNF-α, IL-6, and others (16, 17). The nuclear factor NF-κB pathway plays a vital role in the mediation of the transcriptional expression of proinflammatory genes (18). Normally, the transcription factor NF-κB is sequestered in the cytoplasm bound to its inhibitor IκBα, whose phosphorylation is dependent on IKKβ activity. Once stimulated, the p65 subunit of NF-κB separates from IκB and translocates into the nucleus, where NF-κB can regulate the transcription of several inflammatory cytokine genes, for example TNF-α, IL-6, and IL-1β (4, 19). Therefore, we speculated that the NF-κB pathway was involved in the synthesis of inflammatory cytokines in adipocytes.

Dairy cows with ketosis displayed severe fat mobilization in adipose tissue and high levels of inflammation (3, 14, 20). PLIN1 plays an important role in the homeostasis of adipocytes. We reasoned that PLIN1 might also be involved in the regulation of the NF-κB inflammatory pathway and inflammatory cytokine synthesis. Therefore, the aim of this study was to investigate the role of PLIN1 in lipid metabolism and inflammatory cytokine synthesis in the white adipose tissue of ketotic cows. The results of this study provide a new perspective to elucidate the mechanism of lipid metabolism regulation and inflammation in dairy cows with ketosis.

## Materials and Methods

The Ethics Committee on the Use and Care of Animals at Jilin University approved the study protocol (Changchun, China). All animals used in this study were handled in accordance with the Guiding Principles of Animal Experiments by the Chinese Society of Laboratory Animal Sciences [2015 clinical trial (2015-121)].

### Animals

Lactating Holstein cows with similar numbers of lactation days (median = 3 days, range = 2–4 days) and milk days (median = 6 days, range = 3–9 days) were chosen from a 1,000 cow dairy farm located in Jilin City, Jilin Province, China. Twenty cows were divided into healthy (10 cows) and ketosis (10 cows) groups. The basic descriptions of the ketotic and healthy cows are provided in Table 1. Total mixed ration (TMR) was fed *ad libitum* once per day at 11:00 a.m., which met the animals’ nutritional requirements. The basal diet formulation is shown in Table S1 in Supplementary Material. Cows with ketosis were chosen according to clinical symptoms and the blood concentration of β-hydroxybutyrate (BHB), as described in a previous study (21). Ketosis is defined as a serum BHB concentration higher than 1.2 mM. Blood samples were collected *via* coccygeal venipuncture with heparin before feeding at 5 a.m. and centrifuged at 1,900 *g* for 15 min at 4°C. The blood concentrations of glucose, NEFA, and BHB were detected using a Hitachi 7170 autoanalyzer (Hitachi, Tokyo, Japan; lab assay) with commercially available kits (Randox Laboratories, Crumlin, UK). Subcutaneous white adipose tissue (approximately 3 g) was taken from the tail root by an experienced veterinarian. During the experimental work, the cows were housed in a climate-controlled barn in individual tie stalls to reduce environmental effects.

**Table 1:** The basic description of the clinical ketosis cows and control cows.

	Control (*n* = 10)		Ketosis (*n* = 10)		*P*

	Median	IQR	Median	IQR	
Body weight (kg)	608	593, 629	619	601,637	0.53
Milk production (kg of milk/cow per day)	29.7	26.5, 33.8	25.1	21.3, 27.7	0.0014
BHB (mM)	0.53	0.32, 0.79	3.36	2.86, 4.23	<0.001
NEFA (mM)	0.29	0.17, 0.32	0.88	0.68, 1.10	<0.0001
Glucose (mM)	3.76	3.26, 4.28	2.25	1.96, 2.75	<0.0001

*BHB, β-hydroxybutyrate; NEFA, non-esterified fatty acids*.

*The data are presented as the mean ± SEM*.

### Isolation of Cattle Primary Preadipocytes

The cattle primary preadipocytes were isolated as described in the study of Yin et al. (22). Healthy day old Holstein calves were treated with anesthesia and adipose tissue from the peritoneal omentum and mesentery was obtained by surgery under sterile conditions. The resulting tissue was rinsed in sterile phosphate-buffered saline (PBS) containing penicillin (2,500 U/mL) and streptomycin (2,500 mg/mL) to remove adherent blood. The fascia and blood vessels visible in the tissue were carefully peeled away, the resulting tissue was cut into small pieces of approximately 1 mm^3^, and collagenase type I (Sigma-Aldrich, St. Louis, MO, USA) digestion solution (final concentration 1 mg/mL) was added at 37°C and allowed to incubate for 1.5 h. The mixture was removed from the untreated tissue through a 40 µm cell filter and the filtrate was centrifuged at 1,000 rpm for 10 min. The residual erythrocytes were removed by adding ACK lysis buffer (Beyotime Institute of Biotechnology, Jiangsu, China) into the precipitate and centrifuging at 1,000 rpm for 10 min. The supernatant was discarded, and the pellet was resuspended with basic culture medium (BCM), which was DMEM/F12 with 10% fetal bovine serum and 1% penicillin/streptomycin (P/S). After cell counting, the cell suspension was adjusted to a concentration of 1 × 10^4^/cm^2^ and inoculated in a cell culture flask. The culture was then incubated at 37°C and 5% CO_2_ in a cell incubator for 24 h, and the medium was replaced to remove nonadherent cells and tissue residues. Finally, the BCM was replaced every other day.

### Cell Culture and Treatment

To induce the differentiation of preadipocytes into mature adipocytes, primary cells were seeded in cell culture plates. After cell aggregation was combined to approximately 70%, the BCM was discarded, and the freshly prepared differentiation culture medium 1 (DCM1), which is a final concentration of 0.5 mM IBMX (I-7018 Sigma-Aldrich, St. Louis, MO, USA), 1 µM dexamethasone (D-4902 Sigma-Aldrich, St. Louis, MO, USA), and 1 µg/mL insulin (I-5500 Sigma-Aldrich, St. Louis, MO, USA) in BCM, was used to induce differentiation. After 48 h of culture, DCM1 was discarded and replaced by differentiation culture medium 2 (DCM2), which is a final concentration of 1 µg/mL insulin in BCM, to maintain the differentiation culture. The fresh DCM2 was replaced every other day to keep the culture until visible LDs appeared in the cell, indicating that the cells have completed differentiation. This period lasted approximately 10 days.

Adipocytes were transfected with PLIN1 overexpression adenovirus (Ad-PLIN1), GFP negative control adenovirus (Ad-GFP), the small interfering RNA of PLIN1 (siPLIN1), a negative control of siRNA (NC-siRNA), and 4 µg/mL LPS (Sigma-Aldrich, St. Louis, MO, USA). The detailed grouping is described in the figure legends. The empty adenovirus vectors (Ad-GFP) and PLIN1 overexpression adenovirus (Ad-PLIN1) were constructed by Hanbio (Shanghai, China). The siRNA was designed and synthesized by Gemma Genes (Shanghai, China) based on the bovine PLIN1 mRNA sequence. The siRNA sequence is shown in Table S2 in Supplementary Material.

### Triglyceride Content Determination

After cells were treated in 6-well cell culture plates, the cells were collected by centrifugation after trypsin digestion. The triglycerides were extracted with the tissue triglyceride assay kit (Applygen Technologies Inc., Beijing, China), and the OD value was measured at 550 nm. The triglyceride concentration was calculated according to the standard curve made by the OD values of the standard sample and normalized to the total protein content.

### Oil Red O Staining

After differentiation and treatment of cells in 24-well cell culture plates, 4% paraformaldehyde was added to the cells at room temperature for 15 min. The fixative was discarded and the cells were rinsed three times with PBS to remove the paraformaldehyde. Freshly prepared Oil red O working solution was added to the cells for 30 min of dyeing; the dye was then discarded, and the residual Oil red O was washed away. After adding hematoxylin for 20 min, the dye was washed off with PBS. The LDs were observed and recorded under a microscope.

### RNA Isolation and qRT-PCR

Total RNA was extracted with TRIzol (TaKaRa Biotechnology Co., Ltd., Tokyo, Japan) from adipocytes and adipose tissue, and the concentration was measured by a K5500 micro-spectrophotometer. Total RNA (5 µg) was reverse-transcribed into 20 µL of cDNA with a reverse transcription kit (TaKaRa Biotechnology Co., Ltd., Tokyo, Japan) according to the supplier’s protocol. The primers used were designed by Primer Express software 5.0 and the primer sequences are shown in Table S2 in Supplementary Material. The SYBR green plus reagent kit (Roche, Norwalk, CT, USA) was used to prepare a 20 µL mixture system and gene expression was detected in a 7500 Real-Time PCR System (Applied Biosystems, USA). Gene expression values were normalized with expression values for β-actin (housekeeping gene).

### Western Blot

Total protein of the adipose tissue and adipocytes were extracted using a protein extraction kit (Sangon Biotech Co., Ltd., Shanghai, China) according to the manufacturer’s instructions. The protein concentration was determined using the BCA Protein Assay Kit (Beyotime Institute of Biotechnology, Jiangsu, China). 30 mg of protein was separated using sodium dodecyl sulfate-polyacrylamide gel electrophoresis (SDS-PAGE) with known molecular weight markers (Sangon Biotech Co., Ltd., Shanghai, China). Then, the protein was transferred onto 0.45 µm PVDF membranes. The PVDF membranes were incubated with primary antibodies against p-IκB (Cell Signaling Technology, Inc., Danvers, MA, USA; 1:1,000), IκB (Cell Signaling Technology; 1:1,000), p-NF-κB p65 (Abcam, Cambridge, UK; 1:1,000), NF-κB p65 (Abcam; 1:1,000), sterol regulator element-binding protein-1c (SREBP-1c; Abcam, 1:2,000), PLIN1 (Abcam; 1:1,000), acetyl-CoA carboxylase 1 (ACC1; Abcam, Cambridge, UK), fatty acid synthase (FAS; Cell Signaling Technology; 1:2,000), and ATGL (Abcam, 1:1,000) at 4°C overnight. Subsequently, the PVDF membranes were incubated with an appropriate secondary antibody (Boster, Wuhan, China). Finally, the immunoassay was performed using an enhanced chemiluminescent (ECL) reagent (Pierce Biotechnology Inc., Chicago, IL, USA). Protein gray intensity was quantified by the Gel-Pro Analyzer program normalized to β-actin (Santa Cruz, CA, USA) levels.

### Immunocytofluorescence

Primary preadipocytes were seeded on glass coverslips in a 6-well cell culture plate and induced to differentiate when they were approximately 70% confluent. After differentiation, LPS, siRNA, or adenovirus treatment was performed as described above. Successively, the glass coverslips were washed with 0.01 *M* PBS, fixed with 4% w/v paraformaldehyde for 20 min at room temperature, subjected to antigen recovery with EDTA-Na_2_ (95°C, 5 min) and punched with 0.1% Triton X-100 (Sigma-Aldrich, St. Louis, MO, USA) for 10 min. After washing again, the cells were incubated overnight at 4°C with primary antibody NF-κB p65 (Abcam, Cambridge, UK) diluted 1:100 with goat serum, then treated with goat anti-rabbit IgG conjugated with Cy3 (Beyotime Institute of Biotechnology, Jiangsu, China) at 1:200 in PBS for 30 min at room temperature and re-stained with Hoechst 33258 (Beyotime Institute of Biotechnology, Jiangsu, China). The coverslips were observed and photographed using a laser scanning confocal microscope (Fluoview FV1200, Olympus, Japan).

### Enzyme-Linked Immunosorbent Assay (ELISA)

After differentiation and treatment of cells in 6-well cell culture plates, the supernatant of the culture medium was collected by centrifugation. The levels of IL-1β, IL-6, and TNF-α in the supernatant were measured by an ELISA kit (TNF-α: ml024586; IL-6: ml023908; IL-1β: ml023899; Shanghai Enzyme-linked Biotechnology Co., Ltd., Shanghai, China) according to the manufacturer’s instructions, and the biological replicates were nine in each group.

### Data Analysis

**Figure 1:**
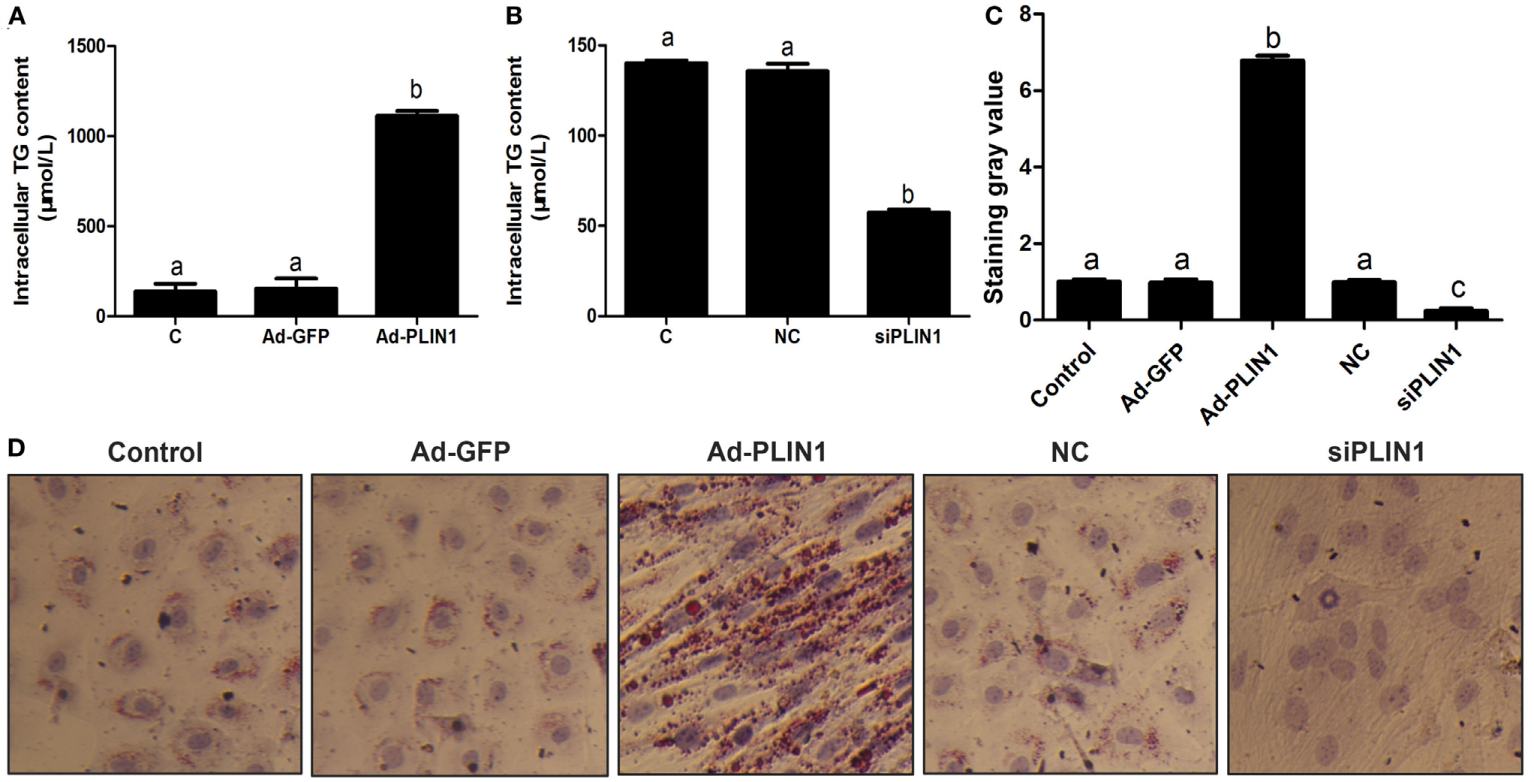
Perilipin 1 (PLIN1) influenced the TAG content in cow adipocytes. Adipocytes were transfected with PLIN1 overexpression adenovirus (Ad-PLIN1), GFP adenovirus (Ad-GFP, a negative control group compared with Ad-PLIN1), small interfering RNA of PLIN1 (siPLIN1), and negative control siRNA. Nine replicate samples were used for each condition (*N* = 9). **(A)** Intracellular TG content in overexpression treatment. **(B)** Intracellular TG content in silencing treatment. **(C)** The Oil red O staining value. **(D)** Oil red O staining results (20×). Abbreviations: C, control group; Ad-GFP, GFP adenovirus; Ad-PLIN1, PLIN1 overexpression adenovirus; NC, negative control of siPLIN1; siPLIN1, small interfering RNA of PLIN1. The data presented are the mean ± SEM. **(A–C)** The same letter indicates no significant difference (*p* > 0.05), whereas different letters indicate a significant difference (*p* < 0.05).

**Figure 2:**
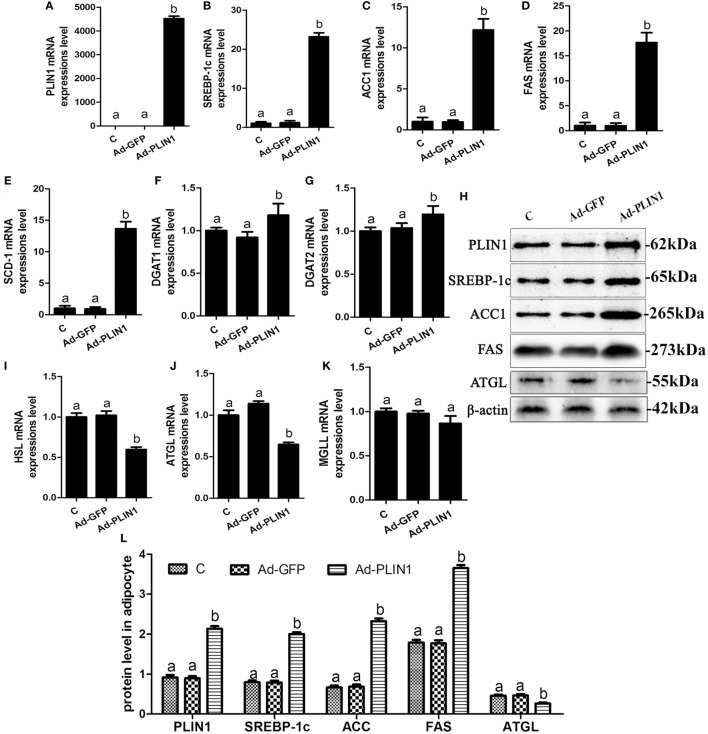
Perilipin 1 (PLIN1) overexpression promoted fatty acids and TG synthesis and decreased TG lipolysis in cow adipocytes. Adipocytes were transfected with Ad-PLIN1 and Ad-GFP. Nine replicate samples were used for each condition (*N* = 9). **(A–G)** The mRNA expression levels of PLIN1 **(A)**, SREBP-1c **(B)**, ACC1 **(C)**, SCD1 **(D)**, FAS **(E)**, DGAT1 **(F)**, and DGAT2 **(G)** in adipocytes. **(H)** Western blot analysis of PLIN1, SREBP-1c, ACC1, SCD1, and FAS. **(I–H)** The mRNA expression levels of hormone-sensitive lipase **(I)**, CGI-58 for adipose triglyceride lipase (ATGL) **(J)**, and MGLL **(K)** in adipocytes. **(L)** The protein expression levels of PLIN1, SREBP-1c, ACC1, FAS, and ATGL. **(C)** Control group. Abbreviations: Ad-GFP, GFP adenovirus; Ad-PLIN1, PLIN1 overexpression adenovirus. The data presented are the mean ± SEM. **(A,B)** The same letter indicates no significant difference (*p* > 0.05), whereas different letters indicate a significant difference (*p* < 0.05).

**Figure 3:**
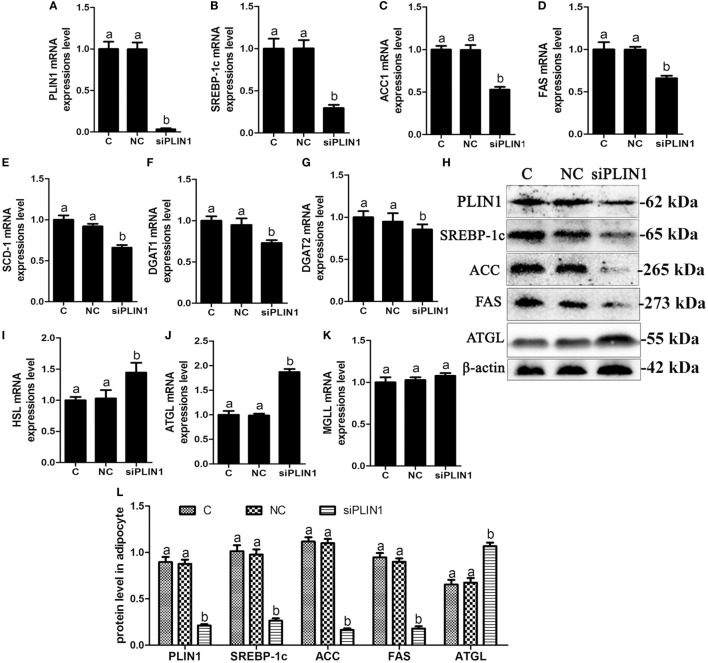
Perilipin-1 (PLIN1) silencing inhibited fatty acids and TG synthesis and increased the TG lipolysis in cow adipocytes. Adipocytes were transfected with siPLIN1 and NC. Nine replicate samples were used for each condition (*N* = 9). **(A–G)** The mRNA expression levels of PLIN1 **(A)**, SREBP-1c **(B)**, ACC1 **(C)**, SCD1 **(D)**, FAS **(E)**, DGAT1 **(F)**, and DGAT2 **(G)** in adipocytes. **(H)** Western blot analysis of PLIN1, SREBP-1c, ACC1, SCD1, and FAS. **(I–H)**: the mRNA expression levels of hormone-sensitive lipase **(I)**, CGI-58 for adipose triglyceride lipase (ATGL) **(J)**, and MGLL **(K)** in adipocytes. **(L)** The protein expression levels of PLIN1, SREBP-1c, ACC1, FAS, and ATGL. **(C)** Control group. NC, negative control siRNA; siPLIN1, small interfering RNA of PLIN1. The data presented are the mean ± SEM. **(A,B)** The same letter indicates no significant difference (*p* > 0.05), whereas different letters indicate a significant difference (*p* < 0.05).

**Figure 4:**
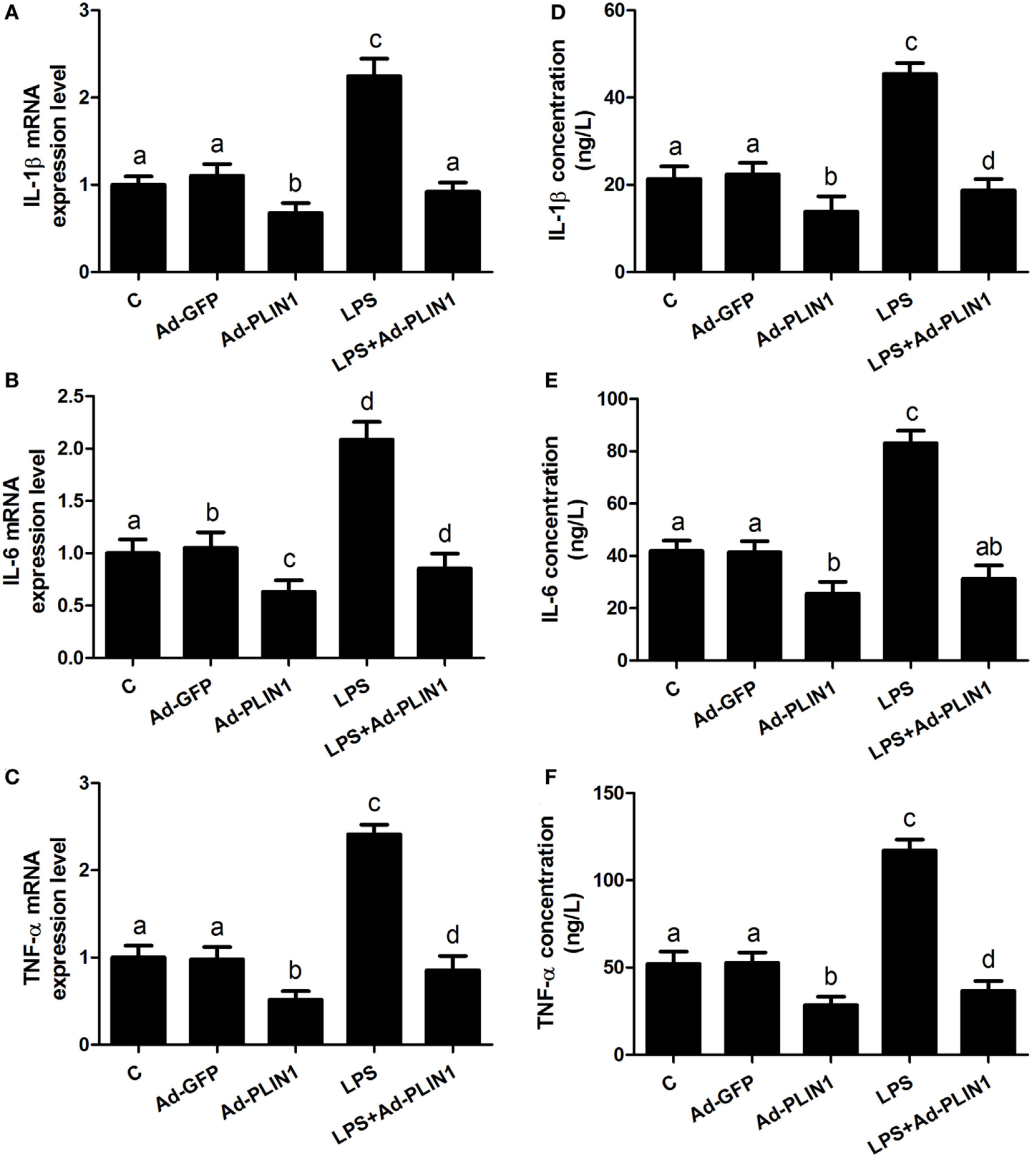
Perilipin-1 (PLIN1) overexpression decreased the LPS-induced expression and synthesis of inflammatory cytokines. Adipocytes were transfected with Ad-PLIN1 and Ad-GFP with or without LPS. Nine replicate samples were used for each condition (*N* = 9). **(A–C)** The mRNA expression levels of IL-1β, IL-6, and TNF-α. **(D–F)** Supernatant concentrations of IL-1β, IL-6, and TNF-α. **(C)** Control group. Abbreviations: Ad-GFP, GFP adenovirus; Ad-PLIN1, PLIN1 overexpression adenovirus; LPS, lipopolysaccharide; LPS + Ad-PLIN1, adipocytes transfected with Ad-PLIN1 and treated with LPS. The data presented are the mean ± SEM. **(A–D)** The same letter indicates no significant difference (*p* > 0.05), whereas different letters indicate a significant difference (*p* < 0.05).

**Figure 5:**
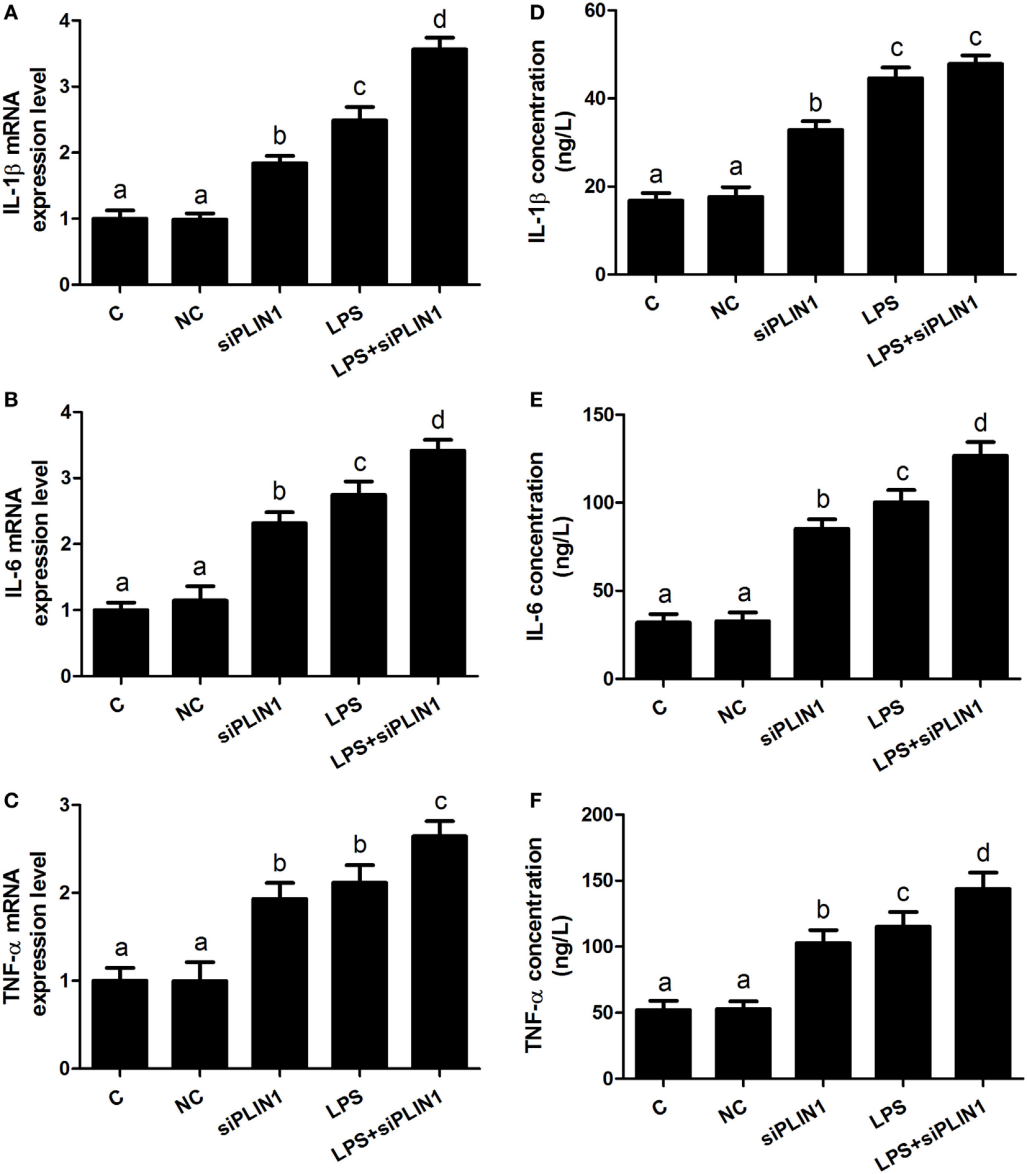
Perilipin-1 (PLIN1) silencing increased the expression and synthesis of inflammatory cytokines. Adipocytes were transfected with siPLIN1 and NC with or without LPS. Nine replicate samples were used for each condition (*N* = 9). **(A–C)** The mRNA expression levels of IL-1β, IL-6, and TNF-α. **(D–F)** Supernatant concentrations of IL-1β, IL-6, and TNF-α. **(C)** Control group. Abbreviations: NC, negative control siRNA; siPLIN1, small interfering RNA of PLIN1; LPS, lipopolysaccharide; LPS + siPLIN1, adipocytes transfected with siPLIN1 and treated with LPS. The data presented are the mean ± SEM. **(A–D)** The same letter indicates no significant difference (*p* > 0.05), whereas different letters indicate a significant difference (*p* < 0.05).

**Figure 6:**
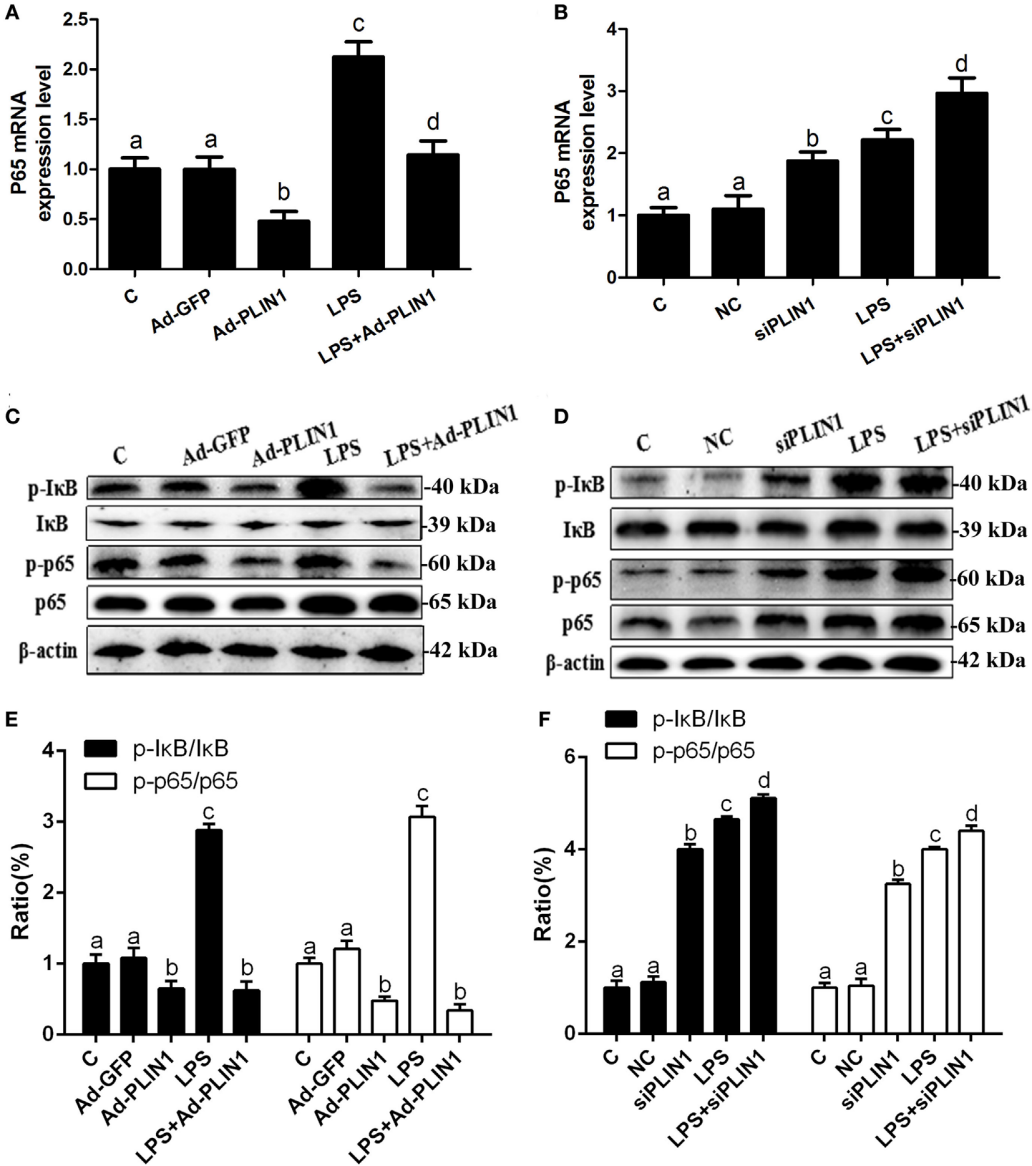
The effect of perilipin-1 (PLIN1) on NF-κB signaling pathway in dairy cow adipocytes. Adipocytes were transfected with Ad-PLIN1, Ad-GFP, siPLIN1, and NC with or without LPS. Nine replicate samples were used for each condition (*N* = 9). **(A,B)** The mRNA expression levels of NF-κB p65. **(C,D)** Western blot analysis of key molecules of the NF-κB signaling pathway in dairy cow adipocytes. **(E,F)** The phosphorylation levels of IκB (p-IκB/IκB) and NF-κB p65 (p-p65/p65). **(C)** Control group. Abbreviations: Ad-GFP, GFP adenovirus; Ad-PLIN1, PLIN1 overexpression adenovirus; LPS, lipopolysaccharide; NC, negative control siRNA; siPLIN1, small interfering RNA of PLIN1. The data presented are the mean ± SEM. **(A–D)** The same letter indicates no significant difference (*p* > 0.05), whereas different letters indicate a significant difference (*p* < 0.05).

**Figure 7:**
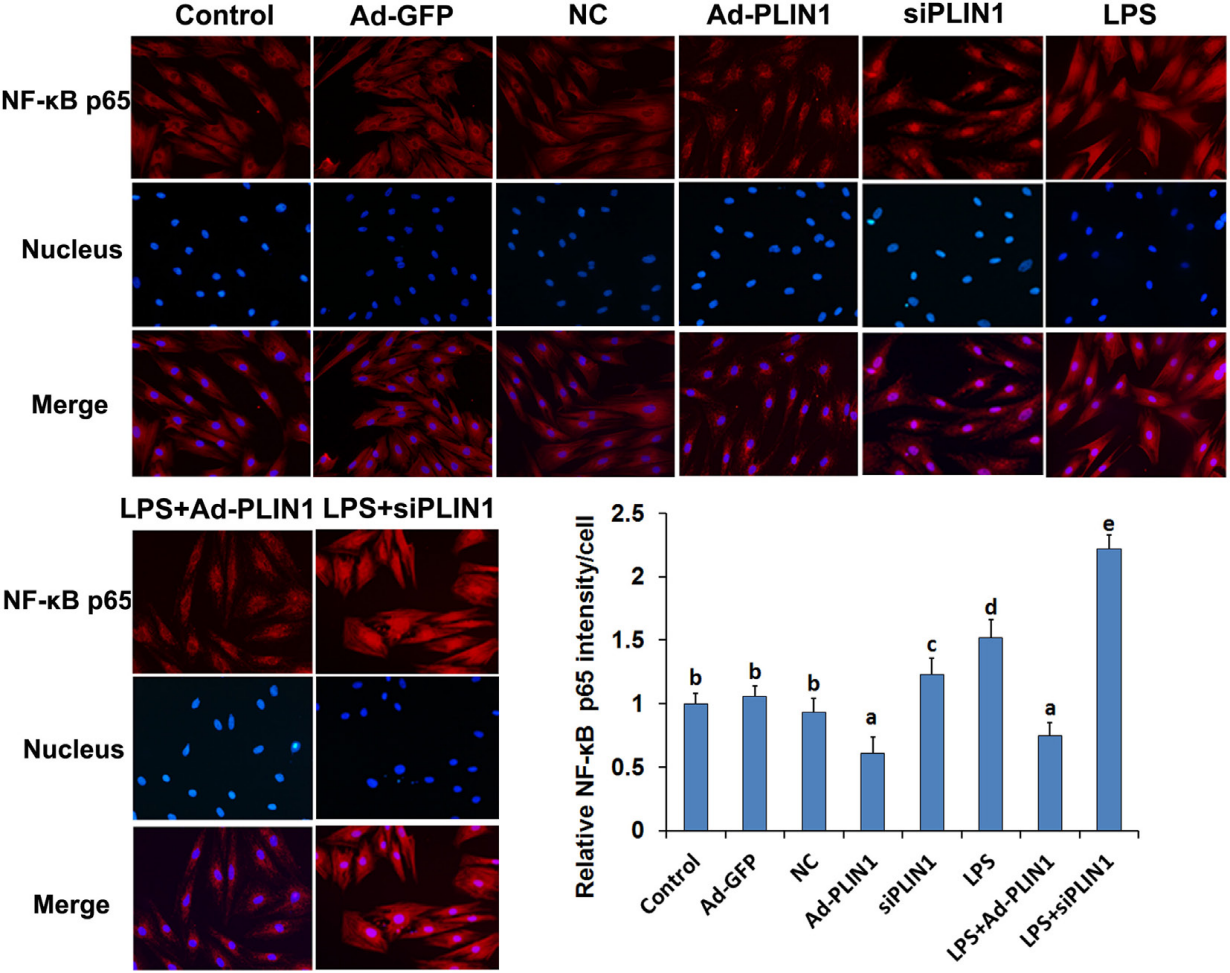
The effect of perilipin-1 (perilipin-1) on nuclear translocation of NF-κB p65 in cow adipocytes. Adipocyte grouping was described as in Figure 6. Nine replicate samples were used for each condition (*N* = 9). The data presented are the mean ± SEM. (a-e) The same letter indicates no significant difference (*p* > 0.05), whereas different letters indicate a significant difference (*p* < 0.05).

The results were expressed as the mean ± SEM. Data in Table 1 were analyzed with a non-parametric test and expressed as median and interquartile ranges. A paired *t*-test was performed to determine the difference between control cows and ketotic cows. The *in vitro* data (Figures 1–7) with more than two groups were tested with ANOVA along with a subsequent Bonferroni correction. All data were analyzed by Statistical Package for the Social Sciences (SPSS) 16.0 software (SPSS Incorporated, Chicago, IL, USA). *P* < 0.05 was considered to be statistically significant compared to the control group.

## Results

### The Effect of PLIN1 on TAG Synthesis in Bovine Adipocytes *In Vitro*

To investigate the effect of PLIN1 on TAG synthesis, bovine adipocytes were transfected with PLIN1 overexpression adenovirus (Ad-PLIN1) or silencing siRNA (siPLIN1), and the intracellular TAG content was detected. As shown in Figure 1A, the TAG content was significantly higher in the Ad-PLIN1 group than in the control group (*p* < 0.05). Conversely, PLIN1 silencing significantly reduced the intracellular TAG (Figure 1B; *p* < 0.05). Furthermore, oil red O staining also showed the same results (Figures 1C,D). There were more and larger LDs in the Ad-PLIN1 group compared to the control group. However, PLIN1 silencing markedly decreased the number and size of LDs in cow adipocytes. These results indicate that PLIN1 can mediate TAG homeostasis in adipocytes.

### The Effect of PLIN1 on Lipid Metabolic Enzymes in Cow Adipocytes

To explore the regulated mechanism of PLIN1 on lipid metabolism in adipocytes, the expression levels of transcription factors and enzymes involved in lipid synthesis and lipolysis were determined. PLIN1 overexpression adenovirus significantly up-regulated the mRNA and protein expression of PLIN1 in adipocytes (Figures 2A,H; *p* < 0.05). This finding indicates that the adenovirus-mediated overexpression of PLIN1 in adipocytes was successful and effective. SREBP-1c is a crucial transcription factor that mediates intracellular lipid synthesis (23). The mRNA expression of SREBP-1c and its target genes ACC1, FAS, and SCD-1 were higher in PLIN1 overexpressed adipocytes than in control group (Figures 2B–E; *p* < 0.05). Furthermore, the mRNA levels of diacylglycerol acyltransferase 1 (DGAT1) and diacylglycerol acyltransferase 2 (DGAT2), involved in TAG synthesis (24), were also markedly augmented (Figures 2F,G; *p* < 0.05). In contrast, the expression of lipolysis genes ATGL and HSL was significantly lower in the PLIN1 overexpressed adipocytes than in control group (Figures 2I,J; *p* < 0.05). However, there was no significant change in the expression of monoacylglycerol lipase (MGL) (Figure 2K; *p* > 0.05) that converted monoacylglycerol (MAG) to fatty acids and glycerol (25). As the changes in mRNA expression, the protein expression levels of SREBP-1c, ACC1, and FAS were significantly up-regulated (Figure 2H, *p* < 0.05), but ATGL was significantly downregulated (Figure 2H, *p* < 0.05). These results indicate that overexpression of PLIN1 can upregulate SREBP-1c-mediated fatty acid and TAG synthesis genes and inhibit lipolysis genes in adipocytes.

To further investigate the role of PLIN1 on lipid metabolism, adipocytes were transfected with PLIN1 silencing siRNA (siPLIN1). The expression level of PLIN1 mRNA in the silenced group was only approximately 3% of that of the control group (Figure 3A; *p* < 0.05). Contrary to the results of PLIN1 overexpression, the mRNA expression of SREBP-1c, ACC1, FAS, SCD-1, DGAT1, and DGAT2 was significantly decreased in siPLIN1-treated adipocytes (Figures 3B–G; *p* < 0.05). However, the mRNA expression of ATGL and HSL showed a reversed result (Figures 3I,G, *p* < 0.05). The mRNA expression of MGLL still showed no significant change (Figure 3K, *p* > 0.05). Furthermore, the protein expression levels of SREBP-1c, ACC1, and FAS were significantly downregulated (Figure 3H, *p* < 0.05), but ATGL was significantly upregulated (Figure 3H, *p* < 0.05). These results suggest that PLIN1 silencing can inhibit SREBP-1c-mediated fatty acid and TAG synthesis genes and upregulate lipolysis genes in adipocytes.

### The Effect of PLIN1 on the Synthesis and Secretion of Inflammatory Factors in Bovine Adipocytes *In Vitro*

We further sought to dissect the links between PLIN1 and inflammatory cytokine synthesis. LPS treatment significantly upregulated the mRNA levels of IL-1β, IL-6, and TNF-α in adipocytes (Figures 4A–C; *p* < 0.05). However, PLIN1 overexpression in adipocytes by adenovirus significantly inhibited the mRNA levels of IL-1β, IL-6, and TNF-α induced by LPS (Figures 4A–C; *p* < 0.05). In addition, these cytokines in culture supernatant were detected using ELISA. PLIN1 overexpression also significantly decreased these cytokines, secreted by adipocytes, induced by LPS (Figures 4D–F; *p* < 0.05). Interestingly, inhibition of PLIN1 by siRNA significantly upregulated the mRNA levels of IL-1β, IL-6, and TNF-α in adipocytes (Figures 5A–C; *p* < 0.05). Furthermore, LPS treatment plus PLIN1 silencing further enhanced the mRNA expression and synthesis of IL-1β, IL-6, and TNF-α compared with the LPS group (Figures 5A–F; *p* < 0.05). Taken together, these results indicate that PLIN1 regulates the expression and synthesis of inflammatory cytokines in cow adipocytes.

### PLIN1 Inhibits the NF-κB Pathway to Regulate the Synthesis and Secretion of Inflammatory Cytokines in Adipocytes

It is known that the NF-κB pathway mediates the expression of IL-1β, IL-6, and TNF-α (18). We tested the effects of PLIN1 on the NF-κB pathway. LPS treatment significantly increased the mRNA level of NF-κB p65 (Figures 6A,B; *p* < 0.05) and phosphorylation levels of IκB (p-IκB/IκB) and NF-κB p65 (p-NF-κB p65/NF-κB p65) (Figures 6C–F; *p* < 0.05) compared with the control group. Nevertheless, PLIN1 overexpression abrogated the induction of the NF-κB inflammatory pathway in response to LPS treatment of adipocytes (Figures 6A,C,E; *p* < 0.05). In contrast to PLIN1 overexpression, PLIN1 silencing significantly increased the mRNA expression of NF-κB p65 (Figure 6B; *p* < 0.05) and the phosphorylation levels of IκB and NF-κB p65 (Figures 6D,F; *p* < 0.05) relative to those of the control group in adipocytes. Furthermore, PLIN1 silencing plus LPS treatment further increased the activation of the NF-κB pathway compared with the LPS group (Figures 6B,D,F; *p* < 0.05). Overall, these results suggest that PLIN1, at least in part, inactivates the NF-κB pathway and inhibits the expression and secretion of inflammatory cytokines in adipocytes, and PLIN1 silencing loses the inhibitory function on the NF-κB pathway.

We next detected the nuclear translocation of NF-κB p65 using immunofluorescence. LPS significantly increased the fluorescence intensity and nuclear translocation of NF-κB p65 in adipocytes (Figures 7A,B; *p* < 0.05), but this effect was abolished when PLIN1 was overexpressed (Figures 7A,B; *p* < 0.05). Conversely, inhibition of PLIN1 by siPLIN1 significantly increased the fluorescence inte nsity and nuclear translocation of NF-κB p65 in adipocytes (Figures 7A,B; *p* < 0.05). Furthermore, inhibition of PLIN1 could further promote the LPS-induced overactivation of NF-κB p65 (Figures 7A,B; *p* < 0.05). These results further suggest that PLIN1 regulates the NF-κB pathway in cow adipocytes.

### The Fat Mobilization and NF-κB Inflammatory Pathway Were Overinduced in the Adipose Tissue of Dairy Cows with Ketosis

**Figure 8:**
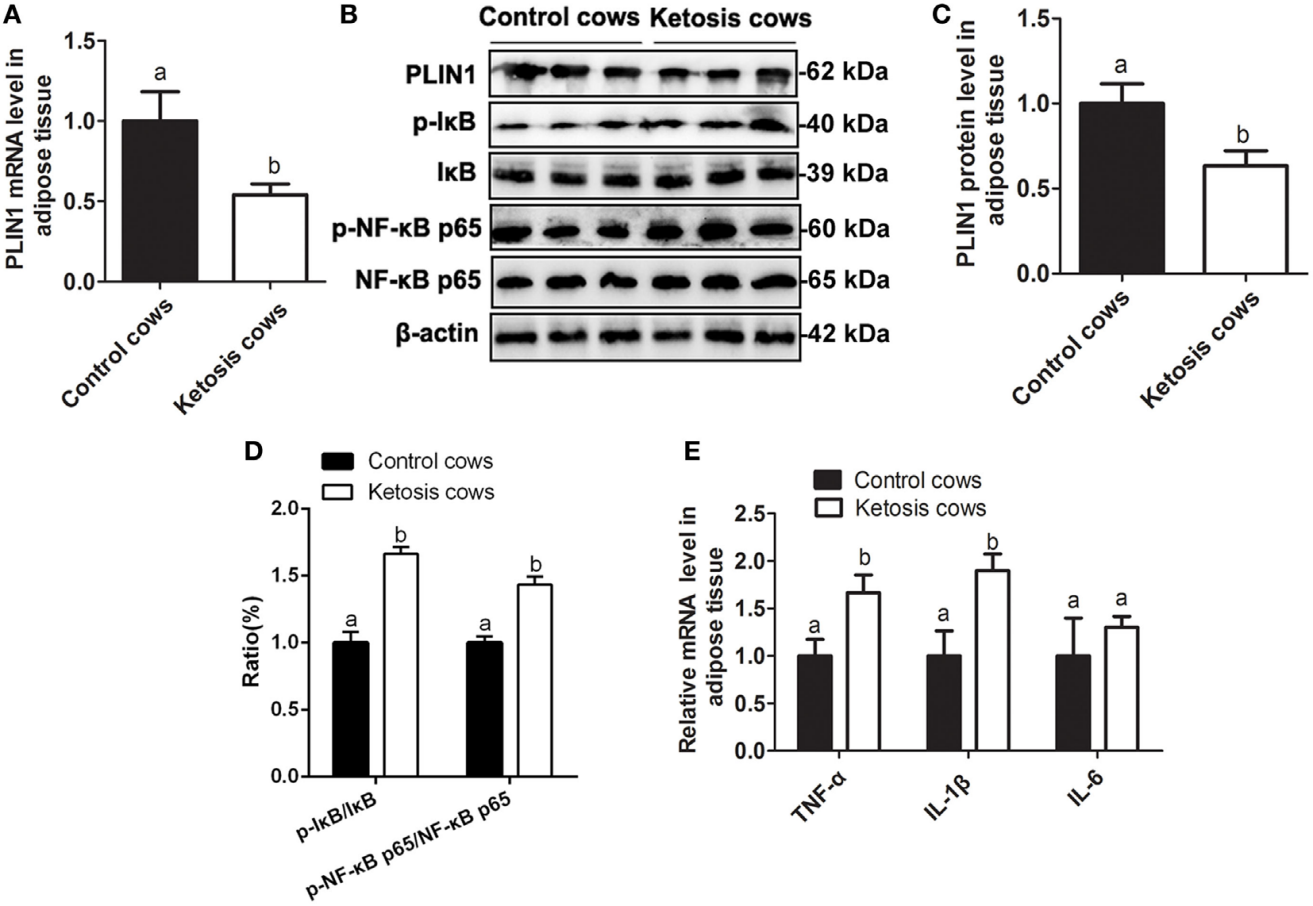
Low expression of perilipin-1 (PLIN1) and overactivation of the NF-κB pathway in the adipose tissue of ketotic cows. **(A)** The mRNA expression level of PLIN1 in the cow adipocytes of control or ketotic cows. **(B)** Western blot analysis of PLIN1 and key molecules of the NF-κB signaling pathway in the cow adipocytes of control or ketotic cows. **(C)** The protein level of PLIN1. **(D)** The phosphorylation levels of IκB (p-IκB/IκB) and NF-κB p65 (p-p65/p65). **(E)** The mRNA expression levels of TNF-α, IL-1β, and IL-6 in adipose tissue of ketotic and control cows. Ten control cows (*N* = 10) and 10 ketosis cows (*N* = 10) were used in this study. The data presented are the mean ± SEM. **(A,B)** The same letter indicates no significant difference (*p* > 0.05), whereas different letters indicate a significant difference (*p* < 0.05).

As shown in Table 1, the blood glucose concentration was significantly lower but NEFA and BHBA concentrations were markedly higher in cows with ketosis than in control cows (*p* < 0.01). This indicates that dairy cows with ketosis display severe NEB and fat mobilization. To test whether our findings in adipocytes are valid in the adipose tissue of dairy cows with ketosis, we measured the expression of PLIN1 and the NF-κB inflammatory pathway in the adipose tissue of dairy cows with ketosis. The mRNA and protein levels of PLIN1 in adipose tissue were markedly lower in ketotic cows than in control cows (Figures 8A–C; *p* < 0.05). The phosphorylation levels of IκB and NF-κB p65 (Figures 8B,D; *p* < 0.05) and mRNA levels of IL-1β and TNF-α (Figure 8E; *p* < 0.05) also significantly higher in ketotic cows than in control cows. Taken together, these data corroborate the conclusion that low expression of PLIN1 increases the lipolysis and overactivates the NF-κB inflammatory pathway in cow adipose tissue.

## Discussion

Perinatal cows with ketosis or fatty liver exhibit severe NEB, which initiates fat mobilization and induces lipid metabolism disorder in cow adipocytes (2, 3). Interestingly, adipose tissue is not only simple lipid storage, but also an endocrine tissue, which can participate in the synthesis and secretion of inflammatory cytokines, including IL-1β, IL-6, and TNF-α (26). Furthermore, the blood concentrations of IL-1β, IL-6, and TNF-α were markedly elevated in cows with fatty liver or ketosis compared with healthy cows (3, 14). As an adipocyte-specific lipid-coated protein, PLIN1 has been shown to be involved in the regulation of adipose lipid synthesis and lipolysis in mice and humans (12, 27). Here, our data also demonstrated that PLIN1 could maintain lipid metabolism homeostasis in cow adipocytes. Importantly, we found that PLIN1 inhibited NF-κB pathway to decrease inflammatory cytokines synthesis in adipocytes.

Lipid droplets are dynamic cellular organelles in adipocytes. PLIN1 is the most abundant protein on adipocyte LDs. PLIN1 acts as a scaffold at the LD surface and are suggested to have a structural and/or regulatory role in LD formation and function (28). Under basal conditions, PLIN1 prevents basal lipolysis and increases lipid synthesis and LD formation (29). PLIN1 knockout increases basal lipolysis, decreases LD size in adipocytes, and causes resistance to diet-induced obesity in mice (30). In this study, TAG determination and Oil red O staining showed that PLIN1 overexpression increased, but PLIN1 silencing inhibited the TAG synthesis and LD formation, consisting with previous studies in mice (11, 29, 30).

Intracellular TAG content is dependent on the balance of lipid synthesis and lipolysis in adipocytes (31). The nuclear transcription factor SREBP-1c is widely involved in the regulation of lipid synthesis through mediating the transcriptional expression of ACC1, FAS, and SCD-1 (23). These three enzymes are all involved in long-chain fatty acid biosynthesis and their expression levels reflect the ability of fatty acid synthesis. We found that PLIN1 overexpression significantly upregulated the expression of SREBP-1c and its target genes in adipocytes, whereas PLIN1 silencing reversed these effects. Takahashi et al. (32) also reported that the expression and activation of SREBP-1 and its target genes (SCD-1, ACC1, and FAS) were significantly increased in the adipose tissues of PLIN1 overexpressed mice, but markedly reduced in PLIN1 knockout mice (32). Our data and a previous study suggested that PLIN1 positively contributed to lipid synthesis. DGAT1 and DGAT2 catalyze the combination of fatty acid acyl-CoA and 3-phosphoglycerine, which is the final step of TAG biosynthesis (24). Interestingly, the expression of DGAT1 and DGAT2 in adipocytes was significantly increased by PLIN1 overexpression, but was markedly decreased by PLIN1 silencing. Taken together, these results indicate that PLIN1 promotes fatty acid and TAG synthesis in cow adipocytes. Furthermore, we found that the effect of PLIN1 on fatty acids synthesis pathway (SREBP-1c, SCD-1, ACC1, and FAS) was stronger than that of TAG synthesis pathway (DGAT1 and DGAT2), which maybe because SCD-1, ACC1, and FAS were the direct target genes of SREBP-1c (23). Although DGAT1 and DGAT2 were not the direct target genes of SREBP-1c, increasing evidence also demonstrated that SREBP-1c could regulate the expression of DGAT1 and DGAT2 (33, 34). Therefore, PLIN1 plays an important role in the fatty acids synthesis in adipocytes.

Maximal activation of adipocyte lipolysis requires three lipases: ATGL, HSL, and MGLL. ATGL hydrolyzes TAG into DAG to release free fatty acid (FFA), while HSL hydrolyzes DAG into monoacylglycerol (MAG), releasing another FFA (22). Finally, MGLL converts MAG to FFA and glycerol (25). PLIN1 restricts ATGL and HSL from LDs and suppresses lipolysis, whereas phosphorylated PLIN1 permits ATGL/CGI-58 and HSL association with LD surfaces to activate lipolysis (35). Our data showed that the expression of HSL and ATGL was significantly downregulated by PLIN1 overexpression, but was markedly upregulated by PLIN1 silencing, suggesting that inhibition of lipolysis by PLIN1 is not only present in the regulation of phosphorylation, but also at the level of gene transcription and protein translation. Overexpression of PLIN1 in 3T3-L1 preadipocytes resulted in a dramatic reduction in lipolytic activity and a significant increase in stored TAG, suggesting that PLIN1 limited the access of lipases to LDs (36). In addition, constitutive overexpression of PLIN1 in cultured adipocytes blocks the ability of TNF-α to increase lipolysis, confirming a role for PLIN1 expression in regulating basal or constitutive lipolysis (6). Studies comparing lipolysis in PLIN1-null and wild type mice revealed that PLIN1-null adipocytes had increased rates of constitutive (unstimulated) lipolysis and reduced catecholamine-stimulated lipolysis (30). These studies further supported the view that PLIN1 in adipocytes blocked lipolysis.

Adipose tissue, as a new endocrine organ, plays an important role in the synthesis of inflammatory cytokines (26). It has been estimated that white adipose tissue contributes approximately 30% of circulating IL-6 in obese humans (37, 38). Furthermore, expression of TNF-α is increased in the white adipose tissue of obese subjects (26). IL-6 and TNF-α are the 2 best-studied cytokines in obesity and have been consistently found to be increased in the serum, white adipose tissue, or both in obese subjects (39). These studies demonstrated that the synthesis of inflammatory cytokines was overinduced in the white adipose tissue of obese subjects. Obese cows are more susceptible to ketosis and fatty liver compared with healthy cows (40). Interestingly, dairy cows with ketosis or fatty liver generally exhibited high levels of inflammation (3, 41). Given the role of PLIN1 on lipid metabolism in adipocytes, PLIN1 might be involved in the regulation of synthesis of inflammatory cytokines. Interestingly, we found that PLIN1 silencing, as a role of LPS treatment, overinduced the activation of NF-κB pathway and increased the expression of inflammatory cytokines. However, PLIN1 overexpression markedly inhibited the NF-κB inflammatory pathway and inflammatory cytokines synthesis. These results indicate that PLIN1 plays an anti-inflammatory role. Importantly, in the adipose tissue of ketotic cows, PLIN1 expression was markedly downregulated, and the NF-κB pathway was overinduced. Low levels of PLIN1 lost the inhibitory effect on the NF-κB pathway and inflammatory cytokine synthesis, which was partly responsible for the high inflammation level in ketotic cows. Additionally, the ketotic cows displayed severe NEB and fat mobilization (2, 3). Low expression of PLIN1 aggravated adipose lipolysis, thereby resulting in the high blood concentration of NEFA in ketotic cows. High levels of NEFA and inflammatory cytokines are the pathologic factors for ketosis (4). Inflammatory cytokines TNF-α and IL-6 could further impair the hepatic lipid metabolism and aggravate the NEFA lipotoxicity such as insulin resistance, mitochondrial dysfunction, and endoplasmic reticulum stress (42–44). These mechanisms interact with each other and collectively promote the development of ketosis. Taken together, our data demonstrated that PLIN1 could mediate lipid metabolism homeostasis and inhibit the NF-κB pathway to decrease inflammatory cytokine synthesis in cow adipocytes.

In summary, low expression of PLIN1 in the adipose tissue of ketotic cows increased the fat mobilization and overinduced inflammatory cytokine expression via the NF-κB pathway. *In vitro*, PLIN1 could mediate lipid homeostasis and inhibit the NF-κB pathway to decrease inflammatory cytokine synthesis in adipocytes. Therefore, PLIN1 may be a valuable target to prevent lipid metabolic disorders and the overproduction of inflammatory cytokines in the adipocytes of cows with ketosis.

## Author Contributions

SZ and XL designed the study and wrote the paper. QZ, QX, and XL reviewed the data, contributed commentary, and edited the manuscript. LL and HD conducted animal experiments, while YL isolated adipocytes. XL performed RT-qPCR and WB analyses. SZ performed Oil red O staining, triglyceride content determination and immunocytofluorescence analysis. All authors reviewed the results and approved the final version of the manuscript.

## Conflict of Interest Statement

The authors declare that they have no conflicts of interest with the contents of this article.

## Abbreviations

NEB, negative energy balance; NEFA, non-esterified fatty acid; LDs, lipid droplets; PLIN1, perilipin 1; HSL, hormone-sensitive lipase; ATGL, CGI-58 for adipose triglyceride lipase; TAG, triglyceride; IL-1β, interleukin 1 beta; IL-6, interleukin 6; TNF-α, tumor necrosis factor alpha; Ad-PLIN1, PLIN1 overexpression adenovirus; Ad-GFP, GFP negative control adenovirus; siPLIN1, small interfering RNA of PLIN1; NC-siRNA, negative control of siRNA; SREBP-1c, sterol regulator element-binding protein-1c; ACC1, acetyl-CoA carboxylase 1; FAS, fatty acid synthase; SCD-1, stearoyl-CoA desaturase 1; DGAT1, diacylglycerol acyltransferase 1; DGAT2, diacylglycerol acyltransferase 2.
