# Supplementary material for: Perilipin 1 Mediates Lipid Metabolism Homeostasis and Inhibits Inflammatory Cytokine Synthesis in Bovine Adipocytes
Source: Front Immunol. 2018 Mar 9;9:467. doi: 10.3389/fimmu.2018.00467 (PMC5854662; doi:10.3389/fimmu.2018.00467)
Supplement: Supplementary file 1 [file table_1.docx]

**Supplementary Table 1** The basal diet formulation %

| Item (%) | Postpartum |
| --- | --- |
| Corn silage | 40.00 |
| Corn | 35.00 |
| Wheat bran | 8.00 |
| Soybean meal | 5.00 |
| Sunflower | 8.00 |
| NaCl | 1.00 |
| Premix* | 1.80 |
| NaHCO_3_ | 1.20 |
| Total | 100.00 |
| Nutrient composition (% of DM) |  |
| NEL (MJ/Kg) | 6.70 |
| CP | 15.20 |
| NDF | 33.45 |
| ADF | 17.20 |
| NFC | 40.40 |
| Ca | 0.70 |
| P | 0.50 |

*The premix provided the following per kg of diets: VA 200,000 IU, VD 70,000 IU, VE 1,000 IU, Fe 2,000 mg, Cu 600 mg, Zn 2,400 mg, Mn 1,300 mg, I 6 mg, Co 7 mg.

DM, Dry Matter; NEL, Net energy for lactation; CP, Crude protein; NDF, Neutral detergent fibre; ADF, Acid detergent fibre; NFC, Non-fibre carbohydrate.

**Supplementary Table 2 The primers sequences of the genes**

| Gene | Primer sequences (5′-3′) |
| --- | --- |
| PLIN1 | For: GATCGCCTCTGAGCTGAAGG |
|  | Rev: AGAGCGGCCCCTAGGATTT |
| SREBP-1c | For: GCAGCCCATTCATCAGCCAGACC |
|  | Rev: CGACACCACCAGCATCAACCACG |
| ACC1 | For: TCCTGCTGCTATTGCTACTCCA |
|  | Rev: CAGTCCCCGCACTCACATAA |
| SCD1 | For: GGCACATCAACTTTACCACG |
|  | Rev: CAGCCACTCTTGTAGCTTTCCTC |
| FAS | For: ACAGCCTCTTCCTGTTTGACG |
|  | Rev: CTCTGCACGATCAGCTCGAC |
| DGAT1 | For: GACCCCTAACCTTTGACCCC |
|  | Rev: CCCAACCTCCCGCTAAGTTT |
| DGAT2 | For: GAAAGGTAGGAGCACGGGTC |
|  | Rev: AGCCCAACACTATTCACGCA |
| HSL | For: TTTCGCACCAGCCACAACCT |
|  | Rev: TCTCATCACCCTCAAAGAAGAGCC |
| ATGL | For: ACGTGGAACATCTCGTTCGC |
|  | Rev: CACCTCGATGATGTTGGCAC |
| MGLL | For: TGAACCTCGTCCTGCCAAAC |
|  | Rev: CTGGTTGCCGAAGCAGACTT |
| IL-1β | For: CTGAACCCATCAACGAAA |
|  | Rev: ATGACCGACACCACCTGC |
| IL-6 | For: AACGAGTGGGTAAAGAACGC |
|  | Rev: CTGACCAGAGGAGGGAATGC |
| TNF-α | For: CTGCCGGACTACCTGGACTAT |
|  | Rev: CCTCACTTCCCTACATCCCTAA |
| β-actin | For: GCCCTGAGGCTCTCTTCCA |
|  | Rev: GCGGATGTCGACGTCACA |
| PLIN1 silence sequence | Sense：GAGAGACACUGCCGAGUAUTT |
|  | Antisense: AUACUCGGCAGUGUCUCUCTT |
